# Distillery Stillage: Characteristics, Treatment, and Valorization

**DOI:** 10.1007/s12010-020-03343-5

**Published:** 2020-06-18

**Authors:** Wioleta Mikucka, Magdalena Zielińska

**Affiliations:** Department of Environmental Biotechnology, University of Warmia and Mazury in Olsztyn, Słoneczna St. 45G, 10-709 Olsztyn, Poland

**Keywords:** Distillery stillage, Valorization, Recovery of bioactive compounds, Polyphenols

## Abstract

Distilleries are among the most polluting industries because ethanol fermentation results in the discharge of large quantities of high-strength liquid effluents with high concentrations of organic matter and nitrogen compounds, low pH, high temperature, dark brown color, and high salinity. The most common method of managing this wastewater (distillery stillage) is to use it for soil conditioning, but this requires thickening the wastewater and may cause soil pollution due to its high nitrogen content. Therefore, treatment of distillery stillage is preferable. This review discusses individual biological and physico-chemical treatment methods and combined technologies. In addition, special attention is paid to valorization of distillery stillage, which is a valuable source of polysaccharides and volatile fatty acids (VFAs), as well as natural antioxidants, including polyphenols and other bioactive compounds of interest to the pharmaceutical, cosmetic, and food industries. New directions in improvement of valorization technologies are highlighted, including the search for new eutectic solvents for extracting these compounds. Such technologies are essential for sustainable development, which requires the use of management and valorization strategies for recovery of valuable compounds with minimal disposal of waste streams.

## Distillery Industry

Alcohol is considered a basic material for a number of industries, e.g., the chemical, pharmaceutical, cosmetics, beverage, food, and perfume industries, and the number of alcohol distilleries is increasing worldwide [1]. In 2005–2007, ethanol production increased by about 41% (from 44.3 to 62.6 billion liters) [2]. The largest producers of bioethanol in the world include developed countries (such as the USA, and in the European Union, Germany, and France) and developing countries (Argentina, China, India, Indonesia, and Malaysia) [3]. In recent years, ethanol production has increased because the European Union has implemented a renewable fuels program, which obliges Member States to use renewable fuels. The share of biofuels from non-food substrates in transport fuels will continue to increase and should reach 10.3% in 2020, 14.0% in 2025, and 19.7% in 2030 [4].

Currently, alcohol production is based on the use of non-waste agricultural substrates, such as cereals, potato starch, molasses from sugar beet, beetroot, and fruits [5]. Ethanol fermentation can be performed with any naturally occurring sugar, starch, or cellulosic material combined with the appropriate pretreatment. Whereas sugary raw materials that contain carbohydrates can be directly assimilated by yeast, starchy raw materials require acid or enzymatic hydrolysis to glucose or fructose before ethanol fermentation [6]. On a global scale, about 47% of ethanol is produced from sugary raw materials [7]. In Europe, Poland is one of the biggest generators of sugar [8], which is produced from sugar beet with a yield of 8.58 t/ha; 2065.3 thousands of tons was produced in 2019/2020 [9]. As a by-product of sugar production, molasses is formed. Molasses contains 30% water and can be stored in tanks for a couple of months [10]. In 2007, 539 million liters of bioethanol from sugar beets and beet molasses was produced in France [5]. A global leader in the production of bioethanol is Brazil. In 2007, 21.3 billion liters of alcohol was produced, using molasses and sugar cane juice [11]. Most distilleries partner with sugar mills and make use of molasses from cane sugar manufacturing as the starting material for alcohol production. Although various biomass materials can be used to prepare ethanol, their usefulness as feedstocks depends on their cost, availability, carbohydrate content, and the ease by which they can be converted into alcohol [12].

Four stages of alcohol production in distilleries embrace feed preparation, fermentation, distillation, and packaging [13]. The feed, or fermentation broth, for ethanol production contains raw materials and nitrogen and phosphate supplements. Fermentation is performed by yeast, for example *Saccharomyces cerevisiae* [14], which convert the carbohydrates into alcohol and then settle in a sludge at the end of the process. The mass after fermentation contains 8–10% ethanol. In a distillation column, the alcohol is separated out as the top product, and the bottom product is a brownish liquid known as distillery stillage [15]. In general, distillery stillage (spent wash) and water for the system cooling are the main contributors in total volume of wastewater produced in particular steps of alcohol production (Table 1).

**Table 1:** Wastewater produced in distilleries

Operations in distilleries	Liter wastewater/liter alcohol	References
Spent wash	11.9	[1]
Fermenter cleaning	1.6	[16]
Fermenter cooling	7.2	[13]
Condenser cooling	7.9	[16]
Floor wash	1.1	[15]
Bottling plant	1.3	[15]
Others	0.8	[16]

Distillery stillage may pose a serious environmental concern since it pollutes the water sources in several ways. First, the dark-colored stillage can block out sunlight, inhibiting photosynthesis and reducing the oxygenation of the water, which is detrimental to aquatic life [17]. Second, a high pollution load causes eutrophication of water bodies. For these reasons, untreated distillery stillage causes depletion of dissolved oxygen in water bodies and harms aquatic flora and fauna [1]. Currently, in some countries, by-products from the distillery industry are used for direct soil fertilization (spreading on the fields and plowing) due to their high content of nitrogen, phosphorus, and organics. As an example, vinasse generated from sugar cane juice fermentation is mainly used as a fertilizer in Brazil [13, 18]. Deep well disposal was another option for disposal of by-products, but due to limited storage capacity and to pollution of underground water, this was not considered a good option.

Due to the production of a large amount of wastewater (12 times as much by volume as the alcohol produced) of very high organic loads [19, 20], distilleries are highly water intensive units. Environmental and economic costs can be optimized through rational management of this effluent. Therefore, according to the principles of a bioeconomy, distillery stillage should be transformed into valuable products [21], like heat, power, or feed and other bioproducts, such as compost [22–24]. For all these reasons, it is not only important to purify distillery wastewater but also to use it as a feedstock for the production of value-added products.

## Characteristics of Distillery Stillage

The pollution of distillery stillage depends on the quality of the substrates and the unit operations used for alcohol production, which means that the characteristics of stillage can differ between distilleries [15, 25]. For every liter of alcohol produced, molasses-based distilleries usually generate about 8–15 L of stillage characterized by high chemical oxygen demand (COD) and biochemical oxygen demand (BOD) [26, 27]. The COD and BOD values of this stillage are due to the presence of a number of organic compounds, such as polysaccharides, proteins, polyphenols, waxes, and melanoidin [28]. Distillery stillage contains about 2% of natural products of sugar and amino acid condensation known as melanoidins [29], which contain a lot of dark brown pigment. These substances lead to environmental pollution [30] and have antioxidant activity, which makes them toxic to microorganisms that are present in wastewater treatment processes [31]. The conventional treatment methods are not enough to eliminate melanoidins [32]. Therefore, treated distillery wastewater still contains almost the same dark brown color as before treatment because of the non-biodegradability of the colored compounds [33]. This is one of the reasons why distillery wastewater is difficult to treat. Distillery stillage has high levels of nutrients, such as nitrogen, phosphorus, potassium, and also of total solids, total dissolved solids, total suspended solids, sulfate, and iron. Table 2 presents the physical and chemical properties of wastewater from the distillery industry [1, 34, 35].

**Table 2:** Physical and chemical characteristics of distillery stillage

Characteristic	Units	Value
pH	–	4.0–4.5
Temperature	°C	71–81
Color	–	Dark brown
Total solids	mg/L	59,000–82,000
Volatile solids	mg/L	38,000–66,000
Total suspended solids	mg/L	2400–5000
COD	mg/L	100,000–150,000
BOD	mg/L	35,000–50,000
VFA	mg/L	2300–2400
Nitrogen	mg/L	1660–4200
Phosphorus	mg/L	225–308
Potassium	mg/L	9600–15,475
Iron	mg/L	1550–1800
Sulfates	mg/L	2100–2300
Calcium	mg/L	2300–2500
Magnesium	mg/L	220–250

The discharge of wastewater produced by distilleries has become increasingly restricted as the requirements of environmental regulations have become more stringent and as understanding grows regarding the negative effects of seasonal discharges of water containing high nutrient and organic loadings into water courses [35]. Numerous environmental directives and regulations have been issued, and laws have been passed to define quality standards for water. To abide by stringent government policies on pollution control, industries have now been forced to look for more effective treatment technologies that are not only environmentally friendly but also cost-effective [36]. Hence, distilleries have faced problems in the management of the wastewater they produce and it has become a challenge to meet the requirements for this wastewater. Treatment of distillery wastewater is difficult and expensive. Many researchers have been investigating technologies for the treatment of distillery wastewater; however, none of these have been found to be effective and economically viable methods of complying with regulations [1].

Distillery stillage can be considered a pollutant, but it has also been used in agriculture. For this application, distillery wastewater is potentially valuable, but it also poses problems and has negative effects on the environment. At high doses (> 250 m^3^/ha), the use of distillery stillage is harmful to plant growth and soil properties, but its application at lower doses (125 m^3^/ha) significantly improves the sprout, growth, and yield of dry land plants, due to its content of nutrients (P, N, K, and Ca). Moreover, the combined application of distillery stillage and natural organic compounds (cattle manure, green leaf manure, and bio-compost) is suitable under dry land conditions [23].

## Treatment of Distillery Stillage

From environmental point of view, elimination of organics from by-products from distillery industry is becoming increasingly important in terms of reduction of soil and water pollution. The treatment of distillery effluents is a challenging issue due to the presence of difficult-to-degrade compounds such as caramel, sugar degradation products, anthocyanins, tannins, and different xenobiotic compounds [25] and their toxic effect on humans and the environment. Such compounds are not effectively removed by conventional biological treatment processes. Hence, several physico-chemical, biological, and integrated treatment processes for effective degradation of organic matter have been explored to address the demands of environmental regulations [18]. The technological methods used for the treatment of distillery stillage are biological (aerobic and anaerobic), physico-chemical (coagulation/flocculation, electrocoagulation, adsorption, advanced oxidation, and membrane processes), and thermal (evaporation/combustion). These methods have been practiced and examined during recent years. The choice of treatment method depends on its efficiency and cost, the type of land where the distillery stillage will be used after treatment, regulatory constraints, and public acceptance of the treatment.

For the complete treatment of distillery stillage, individual methods and technologies should not be used, but instead a comprehensive treatment that involves several sequential technologies should be employed. Such an approach provides multiple benefits in terms of environmental protection, energy conservation, and production of high value compounds. Therefore, in the descriptions below, combined technologies are given in addition to individual processes.

### Biological Treatment

Biological treatment of distillery stillage depends on the natural growth and selection of microorganisms. During this process, the microorganisms utilize pollutants for growth and convert the organic substrates into simpler substances in the presence or absence of oxygen. Both methods (aerobic and anaerobic) can be used separately for the treatment of distillery stillage, but because this kind of wastewater has high concentrations of organic pollutants, in most cases, a combination of both is used.

#### Anaerobic Treatment

Anaerobic digestion is a natural process in which anaerobic microorganisms utilize organic matter and transform it to biogas, which makes this treatment more attractive than aerobic treatment. The process is conducted by a comprehensive ecosystem in which physiologically different groups of microorganisms function and interact with each other. A number of microorganisms are involved in anaerobic digestion, including acetic acid-forming bacteria (acetogens) and methane-forming archaea (methanogens). The anaerobic digestion can treat high-strength distillery stillage more effectively than aerobic treatment because it degrades concentrated wastewater, produces a small amount of sludge, requires less energy, and produces economically valuable bioenergy (biogas) [15]. The by-products from the alcohol industry with a minimum COD concentration of 2800 mg/L can be used in fermenters for biogas production [1]. Furthermore, the anaerobic process is vulnerable to organic load and low pH, and the anaerobic microorganisms grow slowly, which leads to longer hydraulic retention time necessary.

Anaerobic processes in the treatment of distillery stillage have been used in various reactor technologies. UASB reactors have been reported to be particularly successful in the treatment of highly polluted effluents. This technology is attractive because the reactors do not require mechanical mixing and enable recycling of biomass sludge. According to Wolmarans and De Villiers [37], UASB technology not only removed over 90% of COD when treating distillery stillage that contained about 30,000 mg/L of COD but also produced biogas with the productivity of 0.43 m^3^/kg. The process should be carefully controlled by first operating at a low loading rate (4–8 kg COD/(m^3^ day)) to ensure successful startup, and then, when removal efficiency is over 90%, increasing the loading rate up to even 30 kg COD/(m^3^ day) is possible. On the basis of these results, we can deduce that the distillery stillage is a potential source for the production of biogas, and that anaerobic digestion is a reliable solution for the treatment of this type of effluents.

Because anaerobic bacteria are capable of transforming distillery stillage, which is rich in organic compounds, into biogas, anaerobic digestion is highly advantageous for the treatment of such effluent. The process demands less energy input and also very low nutrient load. Generation of energy can reduce operational costs to a large degree as compared with energy-intensive aerobic processes [38].

#### Aerobic Treatment

Although the key disadvantage of the aerobic processes for the treatment of distillery stillage is high energy consumption, these processes are widely used because of their high efficiency and ease of use. They are applied both as a pretreatment and as a final treatment [39].

A large number of microorganisms (bacteria, cyanobacteria, yeast, fungi, etc.) can be used for treatment of distillery stillage in aerobic conditions. Filamentous fungi can be considered important phenolic-degrading organisms, as they frequently grow on wood, utilizing lignin as a carbon source [40, 41]. One of the first publications dealing with the distillery stillage treatment by aerobic methods comes from 1965 [42]. Distillery stillage was treated in a stirred reactor, achieving COD elimination of 60.7%. Other authors used a bioreactor with a fixed bed and recirculation of treated stillage and obtained much higher removal of COD of 98% [17]. The efficiency of treatment depended on the following factors: temperature, pH, COD, and nutrients (ammonia nitrogen and phosphate phosphorus) [7, 19]. For the treatment of distillery stillage coming from maize and rye, the variety of species of *Bacillus* bacteria (thermophilic and mesophilic) was used. In the case of stillage from maize, the removal of COD was 82.6%, but from rye residues, COD elimination was 84.6% [43]. Reports are also available on the aerobic treatment of distillery stillage from wheat grain [19]. The system yielded 64% COD removal at 45 °C and about 90% at 55 °C. Jackson et al. [44] used an aerated bioreactor to treat distillery stillage with a HRT of 14 days. COD was removed from 2255 to  150 mg/L.

Currently, study is focused on the combination of anaerobic and aerobic techniques for effective removal of pollutants from distillery stillage. Kapdan et al. [45] obtained 95% COD and 85% color removal efficiency in anaerobic-aerobic treatment of by-products from the distillery industry. On the other hand, Jimenez et al. [38] investigated the combined aerobic-anaerobic treatment of distillery stillage. The reason for such an order of processes was that phenolic compounds are considered toxic to methanogenic microorganisms and the high salinity can cause osmotic pressure problems to the methanogens. Therefore, in the first step, most of the phenolic compounds, color, and part of COD were removed under aerobic conditions. In the second step, anaerobic treatment eliminated the remaining organic content. Authors achieved COD removal at the level of 96.5%. The time necessary for decomposition of a given organic load, during using combined aerobic-anaerobic treatment, is lower than that necessary for a single treatment. To conclude, combined aerobic and anaerobic treatments lead to more efficient removal of color and COD from distillery stillage than individual conventional biological treatments.

### Physico-chemical Treatment

Physico-chemical methods of distillery stillage treatment combine physical and chemical processes, in which the first process leads to the removal of suspended materials and the second to the elimination of soluble COD. Such processes involve coagulation/flocculation, electrocoagulation, adsorption, advanced oxidation, and membrane treatment [46].

#### Coagulation/Flocculation

Coagulation is a process of agglomeration of suspended particles present in wastewater by inorganic coagulants, such as sulfates and chlorides of iron, aluminum, or copper, or bioflocculants [47]. Prajapati et al. [46] determined the efficiency of removal of COD and color from distillery stillage from a rice grain-based distillery with a COD concentration of 13,600 mg/L using CuSO_4_·5H_2_O at optimum pH of 6. It was shown that this coagulant ensured 91% of COD removal and 85% of color removal. COD and color removal increased as the dosages of coagulant were increased from 20 to 60 mM, but above this limit, increasing the dosage did not increase the percentage of COD removed. The treatment of distillery stillage by coagulation/flocculation led to COD removal of 55, 60, and 72% and color removal of 83, 86, and 92%, respectively, with the use of 60 mM aluminum chloride, 60 mM iron chloride and 30 mL/L of poly-aluminum chloride [48].

Chemical coagulation is an efficient pretreatment process when used before biological or membrane treatment processes or used as a final polishing treatment to eliminate non-biodegradable organic matter from by-products from distillery industry [49]. The disadvantages of coagulation are the high doses of required reagents, the unsatisfactory coagulant recovery rates, and the generation of sludge.

#### Electrocoagulation

Electrocoagulation is a complex, effective alternative to chemical coagulation process for the treatment of COD-rich wastewater. Electrocoagulation is based on the electrolytic dissolution of metals like iron or aluminum and *in situ* production of insoluble suspension which can remove the pollutants from the wastewater by coagulation. In surface complexation mode, the pollutant acts as a ligand to chemically bind hydrous iron or aluminum. In electrocoagulation, direct current is used [50].

Electrochemical treatment of rice grain–based distillery effluent using an iron electrode has been studied by Prajapati and Chaudhari [51] who investigated the effect of pH (3.5–9.5) on COD and color elimination. COD and color removal efficiencies were 83 and 69%, respectively, and the efficiency of organics removal was strongly dependent on the initial pH. Dayaca et al. [52] used electrocoagulation with an aluminum electrode for the removal of COD from distillery stillage, aiming at determination of the optimal current density, distance between electrodes, and pH. The treatment procedure was conducted using a constant electrolysis time of one hour. It was reported that a treatment with current density of 0.2 A/cm^2^, distance between electrodes of 1 cm, and pH of 6 gave the highest COD removal of 79.09%.

This method has severe limitations due to the consumption of large amounts of electricity [53]. Other drawbacks include the production of chemical sludge, which requires additional measures for effective handling, storage, and utilization or disposal [54].

#### Adsorption

Adsorption on activated carbon (AC) is employed for the elimination of color and specific organic pollutants. The efficiency of adsorption technology depends on adsorbent pore volumes, which affect the reactions between the adsorbate and adsorbent, the nature of the adsorbent, and the type of activating agent and activation conditions [55]. AC is regarded an effective adsorbent due to its properties: increased surface area, microporous structure, and high degree of reactivity [13].

Bharagava and Chandra [3] reported decolorization of distillery wastewater that was achieved by using packed bed anaerobic reactor with AC of a surface area of 1400 m^2^/g. Almost complete decolorization was obtained with 70% of the diluted sample. Lalov et al. [56] investigated the treatment of distillery stillage using chitosan as an adsorbent. During 30-min process at an optimum chitosan dosage of 10 g/L, the removal of color and COD at the levels of 98 and 99%, respectively, was observed. Mall and Kumar [57] compared the color removal using AC and bagasse flyash. They noticed about 58% of color reduction with 30 g/L of bagasse flyash and 80.7% with 20 g/L of AC. In addition, the bagasse flyash has high carbon content and the adsorbed organic material further increases its heating value. Therefore, the spent adsorbent can be used for manufacturing fire briquettes.

The main disadvantage of adsorption for the treatment of distillery wastewater is high operational costs [36] and the production of high volume of solid waste [30].

#### Advanced Oxidation

Fenton process, oxidation, ozonation, and wet oxidation are advanced oxidation processes that can be used to treat distillery wastewater. Fenton process is based on generation of hydroxyl radicals (•OH) with extremely high oxidation potential, through catalysis of H_2_O_2_ by Fe^2+^ ion under acidic conditions. The electrochemically active organic compounds react with oxygen, which consequently leads to mineralization of color components and bioresistant fractions present in distillery stillage [51].

Solar photo-Fenton oxidation process showed a significant removal of COD, dissolved organic carbon (DOC), and color by 70.0 ± 3.3, 53.0 ± 3.7, and 75.0 ± 2.2%, respectively [58]. In an electro-Fenton process using iron electrodes for treatment of distillery wastewater, COD and total organic carbon (TOC) removal was at the level of 92.6 and 88.7%, respectively [59].

The highest efficiency of the treatment of distillery stillage with the use of advanced oxidation can be obtained with the combination of these processes with other methods. Ozone oxidation after aerobic treatment of distillery stillage eliminates most of the degradable organic compounds. The un-decomposed phenolic compounds and organic matter remaining after the aerobic treatment can be completely oxidized by ozone [60].

Ramana et al. [61] studied the effects of various operating parameters such as ozone flow rate (5 to 15 L/min), initial pH (2 to 10), current density (0.10 to 0.50 A/dm^2^), and H_2_O_2_ concentration (50 to 500 mg/L) on the removal of COD and color. The results showed that 100% of color and COD removal could be achieved by ozone-electrocoagulation process with an energy consumption of 5.7 kWh/m^3^ during four hours of reaction time. Hadavifar et al. [62] carried out the treatment of distillery wastewater in a hybrid reactor that combined ozone oxidation and adsorption by granular AC. This resulted in 74.23% of COD removal and 68% of color removal at pH of 2. The wastewater treatment by wet-air oxidation process can serve as a pre-treatment step to enhance the biodegradability and facilitate biogas generation in subsequent biological treatment processes [63]. However, these methods are highly energy intensive and hence expensive [61].

#### Membrane Treatment

Membrane processes are considered the most reliable technologies for the treatment of wastewater originating from different industries. These are physical processes that separate the feed stream into permeate and retentate, in which particles are separated on the basis of their molecular size and shape, with the use of specially designed semi-permeable membranes. Although there are a number of different methods of membrane treatment, the most suitable for the treatment of distillery stillage is pressure-driven membrane filtration. The application of microfiltration (MF) and ultrafiltration (UF) for the treatment of distillery wastewater to produce water suitable for reuse in the bioethanol industry has been studied by Vasić et al. [64]. The efficiencies of the MF membrane for the removal of COD, dry matter, total nitrogen, and suspended solids were 80.4, 78.0, 80.4, and 100.0%, respectively. To improve the quality of the effluent, the MF permeate was further filtered through an UF membrane. The efficiencies of removal of the first three pollutants were increased to 90.0, 99.2, and 99.9%, respectively, and the composition of the final permeate indicated that it could be reused in the bio-ethanol production process. Sedimentation and UF were used to treat the winery effluent; from 7,937 mg C/L in the raw effluent, a reduction by 56.6% was obtained in the permeate [65].

For the treatment of distillery effluents, the combination of membrane techniques and biological treatment was used. For example, Satyawali and Balakrishnan [13] operated a membrane bioreactor (MBR) in a continuous mode using submerged 30-μm nylon mesh filters at the organic loading rates from 3.00 to 5.71 kg/(m^3^ day). Up to 41% COD removal and up to 87% suspended solid retention were obtained. Because of high mixed liquor suspended solids of 10–12 g/L and long solids retention time, the low molecular weight compounds were degraded, whereas the high molecular weight compounds comprising the color imparting melanoidins remained unaffected. Similarly, high molecular weight compounds were not degraded when MBR operation was supported by powdered activated carbon (PAC) when treating sugarcane molasses based distillery wastewater [13]. However, organics removal was higher with PAC addition and the operation at higher loading rates (from 4.2 to 6.9 kg/(m^3^ day)) was possible. Moreover, PAC addition increased the critical flux by 23%, which prolonged the period between membrane cleaning.

### Thermal Treatment

The thermal treatment of highly polluted wastewater from distilleries is considered an economical and effective supplement to the anaerobic digestion and oxidation processes. Heating the stillage at high temperatures (160–250 °C) results in large amounts of organic substrate in the form of solid precipitates. This charred solid stillage has a high heating value (17–24 MJ/kg) and could be easily separated by filtration and then dried [66].

#### Evaporation/Combustion

Currently, numerous full-scale plants that use thermal treatment are operated. Combustion of distillery stillage is gaining interest. The treatment relies on the utilization of stillage as a fuel source. Direct combustion of distillery stillage is more effective if the stillage is pre-dried (to less than 55% moisture content). However, the technology is currently expensive, particularly when applied in small to medium size industries. The most prevalent challenge is the high energy requirement for the pre-drying stage and the treatment of toxic gas emissions [67].

The distillery stillage can be dried using hot air (180 °C), which leads to generation of a dry powder (calorific value of around 3200 kcal/kg) [68]. The powder is typically mixed with agricultural waste (20% volume) and burnt in a boiler. Currently, the use of combustion of distillery stillage in fluidized bed has overcome the restrictions caused by the stickiness of distillery stillage and its high sulfate concentration [13]. Combustion is also an effective method of on-site distillery stillage disposal as it is accompanied with the production of potassium-rich ash [69] that can be used for land application.

Table 3 presents the overview of single and combined methods of treatment of distillery residue with the operational conditions and efficiencies of organic compounds and color removal.

**Table 3:** Overview of operational parameters and results of the treatment of distillery residue (OLR—organic loading rate, HRT—hydraulic retention time)

Treatment conditions	Treatment process	OLR (kg COD/(m^3^ day))	HRT (day)	COD removal (%)	BOD removal (%)	Color removal (%)	References
Downflow fixed film reactor	Anaerobic	14.2–20.4	2.5–3.3	60–73	85–97	–	[70]
Upflow anaerobic sludge blanket reactor (25 °C)	Anaerobic	28	–	39–67	80	47	[71]
Upflow anaerobic sludge blanket reactor (30 °C)	Anaerobic	9–11	11–12	70	–	–	[72]
Downflow fluidized bed reactor with ground perlite (25 °C)	Anaerobic	4.5	3.3–1.3	85	–	55	[73]
Granular bed anaerobic baffled reactor (25 °C)	Anaerobic	4.75	4	90–96	80–92	–	[74]
Stirred reactor (35°°C)	Anaerobic	2–4	10.6–53.5	68.6–93.7	–	41	[38]
	Anaerobic after pretreatment with *P. decumbens*	2–4	3.1–15.4	63.5–82.6	–	41	
Stirred-tank reactor (38 °C)	Aerobic	104,000 mg/L (influent COD)	–	85.5	98.6	–	[75]
Reactor with shaking (22 °C)	Aerobic	2–4	5	50.7–52.7	–	41	[38]
Treatment with *P. sajor-caju* (30 °C)	Aerobic	42,000 mg/L (influent COD)	15	82.8	75.3	99.2	[76]
Reactor with shaking (30 °C)	Anaerobic and aerobic	62,000 mg/L (influent COD)	60	88	77.4	80	[77]
Two-stage bioreactor (stage I—alginate immobilized *P. putida*; stage II—batch culture of *Aeromonas* sp. (25 °C)	Anaerobic and aerobic	5	–	66	–	60	[78]
Stirred reactor (23–25 °C) (aluminum coagulant)	Anaerobic and coagulation/flocculation	38,000 mg/L (influent COD)	15	85	–	96	[79]
Upflow anaerobic suspended blanket reactor (iron coagulant)	Anaerobic and coagulation/flocculation	7,800 mg/L (influent COD)	–	87	92	99	[25]
Reactor with flowing oxygen and ozone (30 °C)	Advanced oxidation	59,000–62,000 mg/L (influent COD)	–	79	–	~ 100	[80]
Reactor with ozone-assisted electrocoagulation (30 °C)	Ozonation and electrocoagulation	80,000–90,000 mg/L (influent COD)	–	83 (4 h)	95 (4 h)	~ 100 (2 h)	[81]
Stirred reactor (iron coagulant; potential difference 5 V)	Coagulation/flocculation and electrochemical oxidation	93,650 mg/L (influent COD)	–	95	–	~ 100	[82]
Bioreactor with PAC (21–26 °C)	Adsorption	4.2–6.9	7	68	–	53	[83]
Membrane bioreactor (55 °C)	Membrane treatment	40,000 mg/L (influent COD)	–	94.7	–	98	[84]
Nanofiltration and reverse osmosis (55.2 bar; 25 °C)	Membrane treatment	100,000 mg/L (influent COD)	–	~ 100	94	–	[27]

### Emerging Treatment Technologies

In general, for the processing complex wastewater such as distillery stillage, the integrated application of many techniques is very practical and promising [85]; combining several processes, including thermal integration or advanced oxidation processes, followed by anaerobic digestion as well as adsorption, is still being investigated. In the development of modern technologies of distillery residue treatment, according to the rules of sustainable development, effectiveness and economic points still remains a challenge. Therefore, apart from integrated processes, emerging treatment approaches include improvements of membrane treatment and the use of microalgae.

Despite very high efficiency of organic compounds and color removal, the use of membrane filtration is restricted by membrane cost and high energy consumption for operating the pumps. The greatest effort is put into searching both for new membrane materials that will decrease membrane fouling, thus giving optimal permeate flux and maximal pollutant rejection, and for energy sources that will minimize the cost. For example, Lapišová et al. [86] investigated the use of combinations of ceramic membranes (pore diameter from 0.2 μm to 300 kDa), which are less prone to fouling. Other authors investigated the pretreatment of stillage with the use of natural coagulants to decrease the fouling of MF membrane [87]. In addition, to optimize the operating cost, studies are conducted to increase the efficiency of stillage treatment so that the permeate can be reused further. For example, distillery wastewater may be purified by the combination of UF and reverse osmosis (RO) to obtain high-quality effluent [88]. In addition, to optimize the cost, retentate produced in membrane treatment can be used as an addition to fertilizers or for biogas production [89]. To meet energy demand in the membrane installation, Ryan et al. [90] suggested using power from the anaerobic digester. Therefore, the combination of biological treatment with the membrane filtration (such as MBRs), which favors nutrient and energy recover, seems to be an effective perspective for the development of distillery residue treatment. The promising method of decreasing energy cost may be using solar energy, which is considered the most abundant renewable energy source. However, this solution has only been tested in municipal wastewater treatment plants [91].

Apart from physico-chemical processes of stillage treatment, the use of microalgae seems to be a method that will be developed in the future [92]. This is because microalgae can use waste as a source of nutrients and their by-products can be processed into alcohol, aquaculture feed, poultry feed, and organic fertilizers, thus reducing the total cost of treatment [93, 94]. However, combination of this treatment with other purification methods should be tested to increase the removal of color and odor [95]. Another challenge in the effective application of microalgae in the treatment of distillery residue is the selection of appropriate species of microalgae that effectively remove organic substances [96].

## Recovery of Valuable Products from Distillery Stillage

To valorize distillery stillage, valuable and/or bioactive compounds can be extracted from it. Depending on the raw material used for fermentation, the composition of the stillage varies, as do the substances that can be recovered from them, i.e., polyphenolic compounds, polysaccharides, or volatile fatty acids (VFAs). In the fermented liquor, the content of bioactive substances is influenced by genetic factors, environmental factors, atmospheric conditions, exposure of substrates to stress, and the technological processes used [97]. Therefore, it is important to choose the optimal conditions for the recovery process. The most common techniques of substrate recovery are liquid-liquid extractions and solid-phase extractions [98]. The organic solvents using for recovery of substances are acetone, ethyl acetate, methanol, ethanol, and propanol or their mixtures. From an environmental standpoint, recovery techniques that meet the requirements of green chemistry are increasingly being sought. Therefore, the new alternative green methods and solvents are investigated for the recovery of valuable compounds. One of the options for green technologies could be supercritical fluids where mainly CO_2_ [99] or water [97] are used; however, there are many drawbacks such as restricted range of molecule solubility, high cost of equipment, and production of pure water. Thus, this technology does not offer prospects for the future. Biomass-derived solvents, such as ethyl lactate, glycerol, or 2-methyl tetrahydrofuran, could be used as the next alternative. These solvents are renewable, non-toxic, and not very expensive, but their range of application and solvation properties are limited [100]. Considering the aforementioned statements, finding and developing green solvents, which will increase the efficiency of extraction processes, is a challenge. In conventional solvent extraction, mostly bioethanol is used, which is a biosolvent produced through alcoholic fermentation of sugar or starch-containing food wastes [101]. Extraction that is friendly for the environment should be characterized by reduced energy demands and the use of nontoxic solvents. Moreover, technologies should be characterized by highly effective recovery of substances, which would appear as a promising path towards sustainable production.

### Recovery of Polyphenolic Compounds

Phenolic compounds are commonly found in plants. They have an important role in their growth, development, and reproduction and protect the plants against pathogens and predators. The polyphenol compounds are the most abundant antioxidants and include flavonoids (e.g., flavanols, condensed tannins, anthocyanins, or proanthocyanidins) and nonflavonoids (e.g., phenolic acids, lignins, stilbenes, ellagitannins and gallotannins) [102]. Moreover, polyphenol compounds have anti-inflammatory, analgesic, anticarcinogenic, and antimicrobial (antifungal and antiviral) properties [103]. At high concentrations, however, they are toxic [20]. The toxic effects depend on the concentration of polyphenols and the species of animal. In the case of fish, lethal effects are achieved at relatively low concentrations (5–15 mg/L). For different species, the average LD50 value is 300–600 mg/kg body weight [104]. However, due to the potential health benefits of these compounds, interest in using them in food, cosmetics, and medicine is increasing. Therefore, it could be important to recover phenolic compounds from distillery stillage and control the amount of these compounds that are released into the environment.

The most common method for the recovery of polyphenols is extraction. The main organic solvents for recovery of polyphenols are acetone and ethyl acetate or alkyl alcohols, e.g., methanol, ethanol, and propanol or their mixtures [105]. The final solvent composition is defined by the nature of the polyphenols and the type of sample used. Organic solvents have excellent dissolution abilities. However, they have many disadvantages, such as accumulation in the atmosphere (low boiling temperature), flammability, toxicity, non-biodegradability, and cost [106].

Librán et al. [107] determined the most effective operational conditions (treatment time and concentration of ethanol) of extraction of phenolic compounds from grape marcs. The phenolic compound content and antioxidant properties of the extracts were determined. The highest recovery of phenolic compounds (3.12 mg of gallic acid equivalent/g of grape marcs) was reported during 2 h of extraction in 75% EtOH liquid mixture at pH 2. Longer and shorter extraction times gave lower yield of the process. The best extraction yields were obtained with 75% ethanol solutions because a higher concentration of ethanol led to a decrease in the antioxidant and organoleptic properties of the phenolic compounds.

To increase the efficiency of polyphenol recovery, chemical substances were used to support ethanol extraction. Ross et al. [108] conducted alcoholic extraction of grain using ethanol:water extraction (80:20) at 4 °C for 5 h. They compared the efficiency of the extraction process with three methods of hydrolysis. In the first, alkaline hydrolysis was carried out with 10 M NaOH and then acid hydrolysis was carried out with 12 M HCl. In the second, alkaline hydrolysis was performed with 2 M NaOH, and in the third, acidic hydrolysis was conducted with 12 M HCl. Each of these hydrolysis methods had two variants. In the first variant, 2% ascorbic acid and 13.4 mM EDTA were added. In the second variant, these solutions were not added. The highest yield of phenolic compounds (3.46 mg of gallic acid equivalent/g of grain) was obtained using alkaline hydrolysis with the addition of 2% ascorbic acid and EDTA. These solutions protected the phenolic compounds from decomposition.

In addition, physical processes for increasing the extraction of polyphenols from distillery stillage have been tested. Microwave-assisted extraction (MAE) and ultrasound-assisted extraction (UAE) are gaining increasing acceptance owing to their benign character and the high effectiveness of recovery of various substances. The rapid penetration of microwaves into the solid leads to the generation of heat inside the solid and a rise in temperature. The heating is uniform, which accelerates extraction. UAE offers several advantages such as low cost, versatility, and ease of deployment [109] and may provide similar yields but with a significantly shorter resident time and lower temperature. Brahim et al. [110] investigated MAE of polyphenols from white grape pomace and red marc. They compared the effects of the following reaction conditions: temperature (60–120 °C), residence time (5–20 min), and the concentration of sodium carbonate (0–2.5%) that was used for decreasing degradation of anthocyanidins. With red marc, recovery was most efficient at 100 °C for 8 min without sodium carbonate. With white grape pomace, it was most efficient at 100 °C for 8 min with 2.5% sodium carbonate. The carbonate concentration did not affect phenolic compound extraction to a large extent. Drosou et al. [111] carried out the extraction of red grape pomace. Three different samples were prepared: with air-drying treatment or solar-drying treatment, or without treatment. Untreated and dried samples were extracted using the MAE, UAE, and Soxhlet extraction with water, water:ethanol (1:1), and ethanol as solvents to recover bioactive compounds with high antiradical properties. The drying of grape pomace plays an important role not only in the reduction of the volume of winery waste, but also in the effectiveness of the recovery of bioactive compounds. The highest recovery was in the case of UAE with the use of water:ethanol (1:1) for air-dried grape pomace and solar-dried grape pomace (438,984 and 258,663 ppm gallic acid equivalent in dry extracts, respectively). Moreover, for the untreated grape pomace, the most effective was MAE with the use of water:ethanol (1:1) (219,228 ppm gallic acid equivalent in dry extract).

In the light of recent studies, researchers should focus on extraction with the use of eutectic solvents. Eutectic (low-melting-point) mixtures are liquids formed from natural substances. For example, organic acids (e.g., citric acid), polyols (e.g., glucose), and salts (e.g., choline chloride, sodium citrate) belong to this group. Currently, these liquids attract attention as green extraction media because they are characterized by reduced toxicity, ease of preparation and use, and low cost [112]. UAE of by-products from agriculture was conducted with eutectic solvents as 90% (v/v) aqueous solutions of glycerol:choline chloride (molar ratio of 3:1), glycerol:sodium acetate (3:1), and glycerol:sodium-potassium tartrate:water (5:1:4) [113]. Water and 60% (v/v) aqueous ethanol were used as control solvents. The results showed that the recovery of grape pomace polyphenols with the use of glycerol:choline chloride had the highest efficiency, which was comparable to that of recovery with aqueous ethanol.

The recovery of phenolic compounds from wine residues was also conducted with the sequential processes of centrifugation, MF, UF, and nanofiltration (NF) [114]. This combination allowed the recovery of phenolic products with 45% content of gallic acid equivalents. The antioxidant capacity of the wine residues was analyzed with a 2,2-azino-bis-3-ethylbenzothiazoline-6-sulfonic acid (ABTS) method, and 2 g of Trolox was obtained. This result indicated that the obtained polyphenols could be used as antioxidant products due to their high antiradical properties. The total phenolics and COD were eliminated at the level of 85.1% and 91.8%, respectively. Thus, these processes could serve as a pre-treatment stage for wine residues.

### Recovery of Polysaccharides

Polysaccharides are low-cost materials and have many applications mainly in the food industry. Some function as emulsifiers and surface active agents, and many are used as simple thickeners. They can be classified as viscosity-increasing agents and as gelling agents. Some polysaccharides are used to the preparation of dextran and pullulan and rare sugars [115].

Giacobbo et al. [65] recovered polyphenols and polysaccharides from winery wastewater by sedimentation and UF processes. The sedimentation conditions were optimized by changing the pH from 3.8 to 8.0, while UF was optimized by changing the transmembrane pressure from 0.5 to 4.0 bar and the feed velocity from 0.44 to 0.87 m/s. This process reduced the TOC content by 56.6% whereas the polyphenols and polysaccharides in the retentate have been concentrated by 6 and 5 times, respectively. The concentrations of total polyphenols and total polysaccharides were 7,014 mg/L and 23,892 mg/L, respectively.

Giacobbo et al. [116] investigated the use of aqueous extraction and MF for recovery of polysaccharides and polyphenols from effluents from the first racking of red wine. The most effective combination involved fiftyfold dilution of the solution and MF, which produced a clear permeate. With this combination, 1 g of polysaccharides and 1 g of polyphenols from 1 liter of wastewater were recovered.

### Recovery of VFAs

In addition to biogas production, recovery of VFAs from industrial wastewater is another method of making wastewater treatment economically attractive. These substances can be produced by elimination of the methane-forming phase of anaerobic digestion/fermentation [117]. VFAs are linear short chain (C2-C6) carboxylic acids and functional molecules, which serve as precursors for the sustainable production of added value chemicals (alcohols and aldehydes), polymers and biofuels [118, 119], and polyhydroxyalkanoates [120].

Although VFAs can be produced by processing petrochemicals, this leads to serious negative health and environmental effects. Greenhouse gas emissions from acetic acid production in the petrochemical industry, calculated as CO_2_ equivalent, are 3.3 t/t [121]. Distillery stillage contains sufficient carbon to produce VFAs via acidogenic fermentation, and it is thus a potentially attractive substrate for production of these valuable chemicals. An advantage of acidogenic fermentation is that it can be carried out by mixed cultures, which means that the substrate does not need to be sterilized [122]. To conduct acidogenic fermentation for the production of VFAs, the methanogenic stage of anaerobic digestion can be specifically inhibited by reducing the pH and the solids retention times [122]. The optimum pH for acidogenesis with distillery stillage ranges from 5.5 to 6.5, whereas a pH of 5.0 or less favors the production of alcohol, and a pH of 6.5 or higher favors biogas (methane) production [123]. Therefore, pH must be controlled with great care. The composition of the VFAs that are produced is also substantially affected by retention time [123, 124]; a retention time of 10–12 h has been reported to maximize acid production [125]. Martinez et al. [120] used acidic anaerobic fermentation of red grapes for VFA production. With the use of a batch anaerobic acidogenic wet process, it was possible to recover VFAs from the liquid phase. This process lasted for 16 days, after which 22.2 g/L of total VFAs was obtained; acetic (15.5 g/L) and butyric (4.3 g/L) acids predominated.

Zacharof and Lovitt [126] used membranes to recover VFAs from distillery stillage. MF was carried out to remove solid particles. In MF, 20.74% of the total solid content had been removed (from 15.13 to 11.99 g/L). After this pre-treatment, NF enabled recovery of acetate (53.94 mM) and butyrate (28.38 mM).

Table 4 presents the overview of methods and conditions of recovery of polyphenols, polysaccharides, and VFAs.

**Table 4:** Overview of methods of recovery of polyphenols, polysaccharides, and VFAs

Method	Additional conditions	Type of value-added product	Concentration of value-added product	Reference
Extraction using 53% ethanol aqueous assisted by microwave (100–600 W)	–	Polyphenols	40.35 mg GAE/g dry pomace	[127]
Extraction using 53% ethanol aqueous assisted by ultrasound (20–60 °C)	-	Polyphenols	33.88 mg GAE/g dry pomace	
Extraction using 53% ethanol aqueous assisted by ultrasound (58 °C)	Pretreatment with cellulase	Polyphenols	44.58 mg GAE/g dry pomace	
Ultrafiltration	Aqueous extraction and fiftyfold dilution	Polyphenols	26.4 mg/L of waste	[128]
		Polysaccharides	17.3 mg/L of waste	
Microfiltration	Pre-sedimentation	Polysaccharides	17.89 mg/L	[129]
Anaerobic membrane bioreactor	pH 9; transmembrane pressure 0.5–4.0 bar	Polysaccharides	26.2 mg/L	[130]
	Feed velocity 0.44–0.87 m/s	VFAs	24.0 mg/L	
Batch anaerobic acidogenic wet-process fermentation	–	VFAs	22.20 mg/L (acetic 15.50 mg/L and butyric 4.30 mg/L)	[120]
Extraction using 80% methanol aqueous	–	Polyphenols	28.13 mg/g dry matter	
Supercritical carbon dioxide (SC-CO_2_)	Containing 10% ethanol aqueous	Polyphenols	25.27 mg/g dry matter	

### Challenges in Recovery of Valuable Products from Distillery Stillage

The approach of conversion of organic wastewater, such as distillery stillage, into valuable materials and energy, defines the biorefining concept as one of the elements of sustainable development. This so called waste bio-refinery is in fact a method of waste valorization [21]. However, development of the valorization technology requires the optimization of recovery techniques. So far, distillery stillage has not been tested intensively in terms of the possibility of recovery of polyphenols and other compounds with antioxidant activity. However, based on the recovery results from other wastewater like winery effluents, the recovery techniques will be developed. Currently, attention is being paid to searching for universal, non-toxic, and biodegradable solvents, particularly because natural bioactive compounds can be used in the pharmaceutical, agrochemical, nutraceutical, and cosmetic industry. In this regard, the use of deep eutectic solvents that contain two or more substances started to be tested. They include choline chloride with urea, carbohydrate with urea and chloride salts, decanoic acid with ammonium salts, choline chloride with oxalic acid, etc. Melting point of these solvents is lower than that of single substances and they can replace many conventionally used hazardous solvents in the extraction process [131]. It is known that the composition of eutectic solvents and thus their viscosity, surface tension, and polarity affect the extraction of bioactive compounds, because they influence mass transfer and diffusivity [132, 133]; however, such investigations for distillery residue are still in the future perspective. Studies on these solvents should focus on the selectivity, purity, and stability of the recovered bioactive compounds. The development of recovery using deep eutectic solvents on an industrial scale involves also the possibility of their recycling, which will reduce the process cost.

## Conclusions

Expansion of distillery industry in the world made it necessary to develop effective technologies for the treatment of distillery stillage. Due to the presence of difficult-to-degrade compounds, physico-chemical, biological, and thermal treatment processes, such as aerobic and anaerobic biological treatment, coagulation/flocculation, electrocoagulation, adsorption, advanced oxidation, membrane processes, and evaporation/combustion, have been explored during recent years to address the demands of environmental regulations. It has been documented that for effective degradation of organic matter, particularly in terms of water reuse, a comprehensive treatment that involves several sequential technologies should be employed. Apart from this, much attention is paid to development of membrane treatment and particularly to exploring new membrane materials that will decrease membrane fouling, thus giving optimal permeate flux, to optimizing energy demand and to valorization of retentate.

As it was presented through this review, distillery stillage is a source of valuable substances such as polyphenols, polysaccharides, and VFAs. The methods of their recovery, according to the biorefinery concept, have been overviewed, indicating the need to focus on the selectivity, purity, and stability of the recovered compounds. However, in the light of recent studies, future research should include the searching for biodegradable, reusable, and non-toxic solvents for effective extraction of these bioactive compounds. Hence, the investigations of the use of deep eutectic solvents for valorization of distillery stillage still remains a considerable challenge.
